# Prognostic factors and outcome of patients undergoing hematopoietic stem cell transplantation who are admitted to pediatric intensive care unit

**DOI:** 10.1186/s12887-016-0669-8

**Published:** 2016-08-20

**Authors:** Kang An, Ying Wang, Biru Li, Changying Luo, Jianmin Wang, Chengjuan Luo, Jing Chen

**Affiliations:** 1 Department of PICU, Shanghai Childrenʼs Medical Center, Pudong, Shanghai China; 2 Department of Hematology and Oncology, Shanghai Childrenʼs Medical Center, 1678 Dongfang Road, Pudong, Shanghai China

**Keywords:** Hematopoietic stem cell transplantation, Prognosis, Child, Pediatric intensive care unit

## Abstract

**Background:**

There are many studies about the prognosis and possible predictive factors of mortality for pediatric allogeneic hematopoietic stem cell transplantation (HSCT) recipients requiring pediatric intensive care unit (PICU) treatment, but the related study in China is lacking. This study investigates the data of these special patients in our center.

**Methods:**

This retrospective analysis is based on data from bone marrow center and PICU of our hospital. A total of 302 patients received allogeneic HSCT from January 2000 to December 2012, 29 of them were admitted to PICU because of various complications developed after transplantation. We collected the clinical data, identified the reasons why the patients to PICU, analyzed the mortality of these patients in PICU, and the prognostic factors of these patients.

**Results:**

The main reasons for admission were: respiratory failure (62.07 %), neurological abnormities (13.79 %), renal failure (13.79 %) and others (10.35 %). Twenty-one cases (72.41 %) died. Compared with survivors, the deaths cases had lower pediatric critical illness score (77 vs. 88, *p* = 0.004); higher levels of lactic acid and serum urea nitrogen (4.02 vs. 1.19 mmol/L, *P* = 0.008;11.56 vs. 7.13 m moll /L, *P* = 0.045); more organs damaged (2.05 vs. 1.38, *P* = 0.01), and required more supportive treatments (1.52 vs. 0.63, *P* = 0.02). Univariate analysis identified pediatric critical illness score, use of mechanical ventilation, and the number of supportive treatment as the significant predictors to prognosis. Multivariate analysis by regression showed that pediatric critical illness score was the only independent prognostic factor (*P* = 0.035).

**Conclusions:**

In our study, pediatric allogeneic HSCT recipients who had PICU care had a high rate of mortality. Pediatric critical illness score was the independent prognostic factor for these patients.

## Background

Since the first patient received bone marrow transplantation in 1950, HSCT has progressed to be widely used in a range of malignant or nonmalignant diseases [1]. In 2008, approximately 2,400 children received HSCT in North America. Of those, approximately 90 % of the indication for transplant was malignancy [2, 3]. With the increase of patients who have received HSCT, there has been an increasing necessity for adequate ICU service to manage various transplant related complications. It is reported that about 3.3–35 % of patients receiving HSCT will require ICU admission, and of those requiring ICU admission the mortality ranged from 37–74 % [4–12]. Although there have been many studies abroad, there is limited data from China. Our study aims to investigate the prognosis of HSCT patients in our center, and to elucidate any possible predictors of mortality.

## Methods

### Population

This is a retrospective case–control study. Tertiary PICU. The study cohort consisted of patients less than 18 years old who were admitted to PICU from January 2000 to December 2012 because of various complications developed after allogeneic HSCT. The doctors of BMT and PICU co-managed the patients. All patients' legal guardians signed an informed consent form. The Institutional Review Board at the Shanghai Children’ Medical Center approved the study protocol.

From a chart review, general demographic data, underlying diseases, duration of pre-transplant, transplant type, donor source, and preprocessing scheme (myeloablative, non-myeloablative) were collected. Additionally, all prior post-transplant history was reviewed. Within the first day of admission to PICU, the time from transplant was determined, the pediatric critical illness score (PCIS) was calculated, laboratory tests were completed (complete blood count, hepatic, renal, coagulation studies, and arterial blood gas), and the type of supportive treatment required was documented (mechanical ventilation, vasoactive agents therapy, renal replacement therapy). The duration of mechanical ventilation, length of PICU stay, and outcome of PICU was collected. Additionally, 30-day and 6-month follow up occurred to determine survival at these time intervals.

### Statistical analysis

Continuous variables were compared using the Mann—Whitney *U*-test. Categorical characteristics were assessed by the Fishers-exact test. Correlations between patient characteristics and prognosis were assessed using univariate and multivariate logistic regression models. All tests were two-sided and a *P*-value below 0.05 was regarded as significant. SPSS 17.0 was used for all analyses.

## Results

A total of 302 patients received allogeneic HSCT during the study period. The underline diseases are shown in Table 1. The duration of underline diseases before transplant ranged from 1 month to 37 months (median, 9 months). The demographics and transplant-related data are shown in Table 2.

**Table 1:** Underlying diseases of transplant patients (*n* = 302)

Underlying disease	Number
Malignant diseases:	169
ALL	56
AML	44
MDS	30
CML	27
JMML	9
HPS	2
NHL	1
Nonmalignant diseases:	133
AA	84
PID	
SCID	7
Non-SCID	13
MPS	12
Mediterranean anemia	10
NPD	3
Pure red cell aplasia	2
Fanconi anemia	1
Pyruvate kinase deficiency	1

ALL:acute lymphoblastic leukemia; AML: Acute myeloid leukemia; MDS: myelodysplastic syndromes; CML: chronic myelogenous leukemia; JMML: juvenile myelomonocytic leukemia; HPS: hemophagocytic syndrome; NHL: Non-Hodgkin's Lymphoma; AA: aplastic anemia; PID: primary immunodeficiency diseases; SCID: severe combined immunodeficiency disease; MPS: mucopolysaccharidosis; NPD: Niemaoh-Pickdisease

**Table 2:** Demographics and transplant-related data of patients who were admitted to PICU (*n* = 29)

Variable	Number
Median age (year)	6
Range	0.5-16
Gender	
Male	20
Female	9
Underlying disease	
Malignant:	
AML	5
ALL	4
MDS	3
JMML	1
Burkitt lymphoma	1
CML	1
Non-malignant:	
AA	8
PID-SCI D	5
PID-non-SCID	1
Transplant type:	
Related	11
Unrelated	18
aGVHD(I-IV)	
0-I	20
II-IV	9
cGVHD	0
Preprocessing scheme	
Myeloablative	18
Non-myeloablative	9
No	2
Median time of duration pre-transplant (month)	9
Range	1-37
Median time of duration after completed transplantation (day)	74
Range	2-540

Of these patients, 29 cases made 31 total admissions to PICU during the study period. Twenty-one (72.41 %) died, 8 patients (27.59 %) were discharged from PICU. The 30-day survival rate was 27.59 % (8/29). 6-month survival rate was 24.14 % (7/29).

The most common indication for PICU admission was respiratory failure (18 cases, 62.07 %). Of these patients, 4 cases had cardiovascular failure and 2 had renal failure simultaneously. Four (13.79 %) patients presented with neurological symptoms. Four (13.79 %) patients occurred isolated renal failure. One presented with cardiac arrest. The most common cause of respiratory failure was severe pneumonia (72 %). The most common cause of neurological pathology was central nervous system infection. Twenty patients (68.97 %) required mechanical ventilation. Of those requiring mechanical ventilation, 17 died and 3 were extubated successfully. Thirteen patients received vasoactive agents. Four patients required renal replacement therapy, and 3 of them died.

The comparisons of continuous and categorical clinical variables between survivors and non-survivors are shown in Tables 3 and 4. In their initial lab work, the patients who died had a higher level of lactic acid (4.02 vs. 1.19 mmol/L, *P* = 0.008) and blood uria nitrogen (11.56 vs. 7.13 mmol/L, *P* = 0.045); lower pediatric critical illness score (77 vs. 88, *p* = 0.004), more organs damaged (2.05 vs. 1.38, *P* = 0.01); more supportive therapies (1.52 vs. 0.63, *P* = 0.02), higher incidence of ventilator use (*P* = 0.04) and vasoactive agents use (*P* = 0.04). Univariate analysis (Table 5) identified pediatric critical illness score (PCIS), use of mechanical ventilation, number of supportive therapy as the significant predictors of prognosis. Multivariate analysis by regression (Table 6) showed that PCIS was an independent prognostic factor (*P* = 0.035).

**Table 3:** Comparison of continuous clinical variables between survivors and non-survivors

	Survivor	Non-survivor	Z	*P*
Types of supportive therapies	0.63	1.52	−2.268	0.02
Duration after completed transplantation (day)	84	125.33	−0.098	0.094
PCIS	88	77	−2.792	0.004
Duration of MV (day)	4.88	4.67	−1.227	0.23
Length of PICU (day)	9.5	6.33	−1.720	0.09
Hospitalization time (day)	44.5	28.95	−1.294	0.20
Time course of disease before transplant (months)	16.25	9.76	−1.47	0.15
Number of organs involved	1.38	2.05	−2.450	0.01

**Table 4:** Comparison of clinical categorical variables between survivors and non-survivors

	Survivor	Non-survivor	Chi-square value	*P*
Gender (male/female)	15/6	5/3	0.216	0.68
Underlying disease: malignant/nonmalignant	3/5	12/9	0.895	0.43
Preconditioning: myeloablative/nonmyeloablative	4/4	14/5	2.032	0.34
Donor (related/unrelated)	1/7	10/11	3.035	0.11
aGVHD (Y/N)	3/5	9/12	0.069	1.0
MV (Y/N)	3/5	17/4	5.11	0.04
Renal replacement (Y/N)	1/7	3/18	0.016	1.0
Vasoactive agent (Y/N)	1/7	12/9	4.668	0.04

Y: yes; N: no

**Table 5:** Univariate analysis of prognostic factors

Variable	*P*
Donor (related/unrelated)	0.11
Myeloablative/nonmyeloablative	0.14
Underlying disease (malignant/nonmalignant)	0.35
Gender (male/female)	0.64
Mechanical ventilation	0.03
Pediatric critical illness score	0.02
Respiratory failure	0.50
Renal replacement	0.90
Vasoactive agent	0.05
aGVHD	0.79
BUN	0.19
Lactate	0.07
Types of supportive therapies	0.04

**Table 6:** Multivariate analysis of prognostic factors

Variable	*P*
Mechanical Ventilation	0.06
Pediatric critical illness score	0.04
Vasoactive agent	0.05
Types of supportive therapy	0.05

## Discussion

It is necessary to introduce the current situation of Chinese pediatric HSCT. The pediatric transplantation centers mainly concentrate in Beijing, Shanghai, and Guangzhou in china. Most of the patients were treated by specialized pediatric transplant physicians. A small percentage of patients received HSCT in adult department of Hematology/Oncology. The precise number of pediatric HSCT per year is not clear, however, in the first six month of 2015, the transplantations included by the Chinese HSCT group increase to 5000 cases, there are 800 under 20 years old, so we speculate that pediatric transplantations possibly reach 500 cases. As for our center, from 2001–2005, a total of 31 cases underwent HSCT, from 2006–2010, 140 patients underwent HSCT, from 2011–2014, 321 cases underwent HSCT. With the increase of patients who have received HSCT, there has been an increasing necessity for adequate P ICU service to manage various transplant related complications. The supportive treatments provided include: laminar flow, continuous renal replacement therapy, peritoneal dialysis, noninvasive mechanical ventilation, invasive mechanical ventilation, and central venous pressure monitoring.

In this study we analyzed the outcome and prognostic factors of these special patients in our center. The most common indication for PICU admission was respiratory failure; the most common cause of respiratory failure was pneumonia, followed by diffuse alveolar hemorrhage. Previous studies also demonstrated that respiratory failure was the main indication for admission [13, 14]. Similar studies showed the most common cause of respiratory failure was pneumonia (41 %, 43 %), followed by diffuse alveolar hemorrhage (37 %, 29 %) [15, 16]. Fungal and viral infections were common in these patients [16, 17]. Recent studies have suggested that the effective use of non-invasive ventilation as early as possible can reduce mortality [18, 19].

The other main reasons for admission to PICU were neurological abnormalities and renal failure. The most common cause of neurological abnormalities was central nervous system infection. For children who had renal failure, the mortality rate was as high as 75 %. Previously described common causes of renal failure in this patient population were fluid overload due to hyper hydration, intravenous antibiotics, and renal toxicity induced by conditioning regimen [20]. Aggressive use of diuretics and early initiation of renal replacement therapy can prevent further deterioration of fluid overload, and improve the prognosis of these children [21].

The proportion of patients requiring mechanical ventilation and/or vasoactive agents was significantly higher in non-survivor group. Our analysis showed that gender, underlying disease, conditioning regimen, donor type, and presence of graft-versus-host disease had no significant difference between the two groups.

Previous studies have reported prognostic risk factors including: pulmonary infection, respiratory failure, multiple organ failure (especially pulmonary failure and neurological deterioration), mechanical ventilation, and vasoactive agent support [6, 12, 15, 22]. Our study showed that mechanical ventilation (*P* = 0.03), pediatric critical illness score (*P* = 0.02), the number of supportive therapies (*P* = 0.04) were risk factors for death. Although not significant at the *P* = 0.05 level, it is also notable that the use of vasoactive agents may also be predictive (*P* = 0.053). Multivariate analysis by regression showed that PCIS was the only independent prognostic factor (*P* = 0.035).

PCIS is a widely used scoring system in China. The emergency group of Chinese pediatric society, Chinese medical association drew up it in 1995. The evaluated objects, in addition to neonate, can be divided into two groups according to age (≥1 year old or < 1 year old). Ten physiological indexes are enrolled: heart rate, systolic blood pressure, spontaneous breath rate, oxygen partial pressure under breathing room air, PH value of arterial blood gas, serum sodium, potassium, creatinine or urea nitrogen, hemoglobin, gastrointestinal system condition (stress ulcer hemorrhage and intestinal paralysis, only stress ulcer hemorrhage or other). The scoring criteria is in accordance with the prescribed scope or situation for each item, and then calculate the sum, if there are more than one result of some indexes in the same day, choose the most abnormal one. Total score is 100, if the patients’ score >80, were divided into non-critical group, between 71 ~ 80, were critical group, 70 or less were extremely critical group. The pediatric emergency group had organized two large-scale clinical test of this scoring system; the results verified its usefulness in assessment of the severity of disease. Some researchers evaluated the relationship between the Pediatric risk of mortality score (PRISM) and PCIS, the results showed that these two scoring system has good correlation. So we think that PCIS is an objective, convenient test adapts to a rapid assessment of pediatric critical ill patients. In this study when the PCIS was less than 90, for every 10 points the score was below 90 the odds ratio of death increased 4.2 times (OR = 4.24, 95 % CI 1.30-13.79).

## Conclusions

Pediatric allogeneic hematopoietic stem cell transplantation recipients who requiring PICU treatment had high mortality. The PCIS was an independent prognostic factor for these patients.

## Abbreviations

AA, aplastic anemia; ALL, acute lymphoblastic leukemia; AML, acute myeloid leukemia; CML, chronic myelogenous leukemia; GVHD, graft-versus-host disease; HPS, Hemophagocytic syndrome; HSCT, hematopoietic stem cell transplantation; JMML, juvenile myelomonocytic leukemia; MDS, myelodysplastic syndromes; MPS, mucopolysaccharidosis; NHL, Non-Hodgkin's Lymphoma; NPD, Niemaoh-Pick disease; PICS, pediatric critical illness score; PICU, pediatric intensive care unit; PID, primary immunodeficiency diseases; PRISM, Pediatric risk of mortality score; SCID, severe combined immunodeficiency disease

## References

[1] Thomas ED, Lochte HL, Lu WC (1957). Intravenous infusion of bone marrow in patients receiving radiation and chemotherapy. N Engl J Med.

[2] Pasquini MC, Wang Z. Current use and outcome of hematopoietic stem cell transplantation: CIBMTR summary slide #12, 2010. [Accessed Jan 5 2011].

[3] Growth A, Baldomero H, Aljurf M (2010). Hematopoietic stem cell transplantation: a global perspective. JAMA.

[4] Tomaske M, Bosk A, Eyrich M (2003). Risks of mortality in children admitted to the pediatric intensive care unit after haematopoietic stem cell transplantation. Br J Haematol.

[5] Gilli K, Remberger M, Hjelmqvist H (2010). Sequential Organ Failure Assessment predicts the outcome of SCT recipients admitted to intensive care unit. Bone Marrow Transplant.

[6] Trinkaus MA, Lapinsky SE, Crump M (2009). Predictors of mortality in patients undergoing autologous hematopoietic cell transplantation admitted to the intensive care unit. Bone Marrow Transplant.

[7] Cole TS, Johnstone IC, Pearce MS (2012). Outcome of children requiring intensive care following haematopoietic SCT for primary immunodeficiency and other non-malignant disorders. Bone Marrow Transplant.

[8] Tamburro RF, Barfield RC, Shaffer ML (2008). Changes in outcomes (1996–2004) for pediatric oncology and hematopoietic stem cell transplant patientsrequiring invasive mechanical ventilation. Pediatr Crit Care Med.

[9] Khassawneh BY, White P, Anaissie EJ (2002). Outcome from mechanical ventilation after autologous peripheral blood stem cell transplantation. Chest.

[10] Chima RS, Daniels RC, Kim MO (2012). Improved outcomes for stem cell transplant recipients requiring pediatric intensive care. Pediatr Crit Care Med.

[11] Jacobe SJ, Hassan A, Veys P (2003). Outcome of children requiring admission to an intensive care unit after bone marrow transplantation. Crit Care Med.

[12] Rossi R, Shemie SD, Calderwood S (1999). Prognosis of pediatric bone marrow transplant recipients requiring mechanical ventilation. Crit Care Med.

[13] Keenan HT, Bratton SL, Martin LD (2000). Outcome of children who require mechanical ventilatory support after bone marrow transplantation. Crit Care Med.

[14] Jackson SR, Tweeddale MG, Barnett MJ (1998). Admission of bone marrow transplant recipients to the intensive care unit: outcome, survival and prognostic factors. Bone Marrow Transplant.

[15] Huaringa AJ, Leyva FJ, Giralt SA (2000). Outcome of bone marrow transplantation patients requiring mechanical ventilation. Crit Care Med.

[16] Bojko T, Notterman DA, Greenwald BM (1995). Acute hypoxemic respiratory failure in children following bone marrow transplantation:an outcome and pathologic study. Crit Care Med.

[17] Neofytos D, Horn D, Anaissie E (2009). Epidemiology and outcome of invasive fungal infection in adult hematopoietic stem cell transplant recipients:analysis of Multicenter Prospective Antifungal Therapy (PATH) Alliance registry. Clin Infect Dis.

[18] Hilbert G, Gruson D, Vargas F (2001). Noninvasive ventilation in immunosuppressed patients with pulmonary infiltrates, fever, and acute respiratory failure. N Engl J Med.

[19] Wermke M, Schiemanck S, Höffken G (2012). Respiratory failure in patients undergoing allogeneic hematopoietic SCT--a randomized trial on early non-invasiveventilation based on standard care hematology wards. Bone Marrow Transplant.

[20] Elbahlawan L, West NK, Avent Y (2010). Impact of continuous renal replacementtherapy on oxygenation in children with acute lung injury after allogeneic hematopoietic stem cell transplantation. Pediatr Blood Cancer.

[21] Michael M, Kuehnle I, Goldstein SL (2004). Fluid overload and acute renal failure in pediatric stem cell transplant patients. Pediatr Nephrol.

[22] Diaz MA, Vicent MG, Prudencio M (2002). Predicting factors for admission to an intensive care unit and clinical outcome in pediatric patients receiving hematopoietic stem cell transplantation. Haematologica.

